# Mycotoxin and Gut Microbiota Interactions

**DOI:** 10.3390/toxins12120769

**Published:** 2020-12-04

**Authors:** Philippe Guerre

**Affiliations:** Ecole Nationale Vétérinaire de Toulouse, Université de Toulouse, ENVT, F-31076 Toulouse, France; philippe.guerre@envt.fr

**Keywords:** mycotoxins, gut microbiota, biotransformation, adsorption, health

## Abstract

The interactions between mycotoxins and gut microbiota were discovered early in animals and explained part of the differences in susceptibility to mycotoxins among species. Isolation of microbes present in the gut responsible for biotransformation of mycotoxins into less toxic metabolites and for binding mycotoxins led to the development of probiotics, enzymes, and cell extracts that are used to prevent mycotoxin toxicity in animals. More recently, bioactivation of mycotoxins into toxic compounds, notably through the hydrolysis of masked mycotoxins, revealed that the health benefits of the effect of the gut microbiota on mycotoxins can vary strongly depending on the mycotoxin and the microbe concerned. Interactions between mycotoxins and gut microbiota can also be observed through the effect of mycotoxins on the gut microbiota. Changes of gut microbiota secondary to mycotoxin exposure may be the consequence of the antimicrobial properties of mycotoxins or the toxic effect of mycotoxins on epithelial and immune cells in the gut, and liberation of antimicrobial peptides by these cells. Whatever the mechanism involved, exposure to mycotoxins leads to changes in the gut microbiota composition at the phylum, genus, and species level. These changes can lead to disruption of the gut barrier function and bacterial translocation. Changes in the gut microbiota composition can also modulate the toxicity of toxic compounds, such as bacterial toxins and of mycotoxins themselves. A last consequence for health of the change in the gut microbiota secondary to exposure to mycotoxins is suspected through variations observed in the amount and composition of the volatile fatty acids and sphingolipids that are normally present in the digesta, and that can contribute to the occurrence of chronic diseases in human. The purpose of this work is to review what is known about mycotoxin and gut microbiota interactions, the mechanisms involved in these interactions, and their practical application, and to identify knowledge gaps and future research needs.

## 1. Introduction

Mycotoxins are secondary metabolites produced by fungi, the most abundant of which belong to the genera *Aspergillus*, *Penicillium*, and *Fusarium*. The conditions required for fungal development and the production of mycotoxins vary strongly with the substrate on which the fungal species develop. Climate conditions also result in marked differences in the occurrence of mycotoxins in human food and animal feed, depending not only on the geographic location but also on the year of the study [1]. Because of the diversity of their origin, mycotoxins differ in their structure, leading to marked differences in their physical-chemical and biological properties. Consequently, the toxic effects of mycotoxins vary greatly with the compound studied but also with the animal species [2,3]. Acute exposure to high doses is usually responsible for well-characterized diseases, while sub-chronic and chronic exposure to low doses have been less well characterized but are considered as being responsible for reduced performance, for the reduced ability of the organism to defend itself against pathogens, and more generally for many of the causes of damaged heath. Regulatory guidelines and recommended levels of mycotoxins in food and feed are available in different countries [4,5,6,7]. 

The term gut microbiome refers to all the microorganisms, bacteria, viruses, protozoa, and fungi residing in the gastrointestinal tract. In humans, it is primarily comprised of four main phyla Firmicutes, Bacteriodetes, Actinobacteria, and Proteobacteria [8]. These phyla may also be present, along with others, in the animal gut microbiota, but variations depending on the segment of the gut analyzed have been reported in mouse, rat, pig, broilers, and dairy cows [9,10,11,12,13]. The role of gut microbiota in the degradation of macromolecules; in nutrient and mineral absorption; the synthesis of enzymes, vitamins, and amino acids; and in the production of short-chain fatty acids has long been known. More recently, microbiota–host interactions have also been demonstrated in metabolic diseases, immunity, and neuroendocrine responses [14].

**Figure 1:**
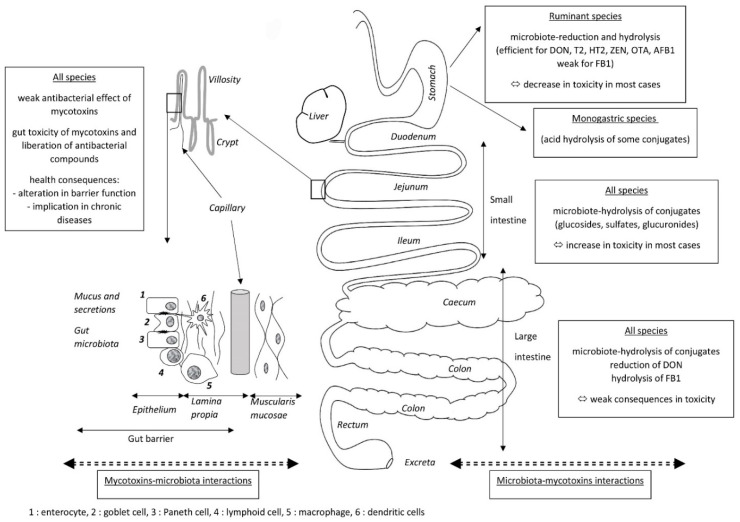
Mycotoxin and gut microbiota interactions.

Because mycotoxins are mainly present in food and feed, the gut is the first target for mycotoxin toxicity, but it is also the site of the absorption of mycotoxins that leads to systemic exposure to these compounds. There are thus many reasons to study mycotoxin and gut microbiota interactions (Figure 1). The first, which concerns the effects of the gut microbiota on mycotoxins, is the ability of the microbiome to modify the toxicity of mycotoxins. These modifications can be caused by several mechanisms, most of which correspond to changes in the toxicokinetics of the mycotoxins. The most frequent mechanism is chemical transformation of mycotoxins by enzymes present in the microbe cells, or excreted by the microbe cells into the gut. The toxic consequences of these transformations vary depending on the metabolites formed. Most of the reactions involved correspond to the hydrolysis of mycotoxins and the formation of metabolites that are less toxic than the parent compound. Cases of activation into compounds that are more toxic than the parent compound are also known, notably the activation of “masked mycotoxins” by hydrolases before their absorption. Another effect of the gut microbiota on the toxicity of mycotoxins is the ability of some constituents of the microbes, mainly the cell wall, to “bind” mycotoxins, thereby reducing their absorption. The interest of studying mycotoxin and gut microbiota interactions is also studying the effects of mycotoxins on gut microbiota, which correspond to the ability of mycotoxins to alter the composition of the gut microbiota. These alterations can be due to direct effects of mycotoxins on the microbes because of their antimicrobial properties, but indirect effects secondary to the toxic effect of mycotoxins on the cells present in the gut can also occur. Indeed, the toxic effects of mycotoxins on cells increase mucus and gut secretions, and diapedeses of cells of the immune system, leading to marked changes in chime composition as well as in the environment in which the microbes grow. Whatever the mechanism involved in the effect of mycotoxins on gut microbiota, its consequence changes the population equilibrium, which can lead to dysbiosis. Changes in the health status corresponding to these alterations can be responsible for bacterial translocation and for the onset of infectious diseases. Dysbiosis is also considered to play a key role in several chronic human diseases, including colorectal cancer, diabetes, and degenerative diseases of the nervous system.

The objective of this review is to describe known mycotoxin–gut microbiota interactions, mainly by focusing on the mechanisms behind these interactions. The first section is devoted to the effects of the gut microbiome on mycotoxins, the second section reviews the effects of mycotoxins on the gut microbiome, and the third section reviews the health consequences of alterations to the gut microbiome following exposure to mycotoxins. The gut toxicity of mycotoxins is not reviewed here, as very recent reviews of the literature are available on this topic [15,16]. 

## 2. Effects of the Gut Microbiota on Mycotoxins

The effects of the gut microbiota on mycotoxins correspond to changes in the toxicokinetic profile of mycotoxins. Two kind of effects are observed, separately or combined, one corresponding to changes in the structure of mycotoxins, i.e., biotransformation, the other corresponding to changes in the absorption of mycotoxins. 

### 2.1. Biotransformation of Mycotoxins

As a very recent review of the literature on the biological degradation of mycotoxins exists [17], we focus here on the biotransformation of mycotoxins that involve the gut microbiota, including those corresponding to the formation of compounds that are more toxic than the parent compound.

#### 2.1.1. Degradation of Mycotoxins

**Figure 2:**
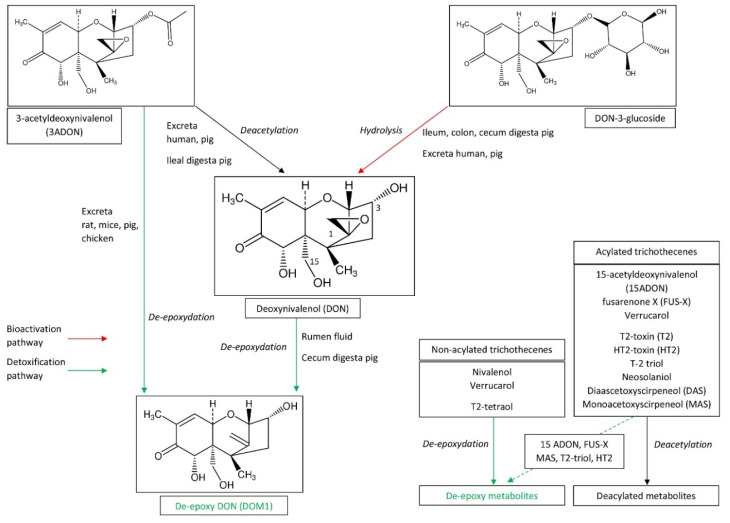
Biotransformation of trichothecenes by the gut microbiota.

Effects of gut microbiomes on mycotoxin toxicity were demonstrated in ruminant species as early as the 1980s [18,19,20,21,22]. From these observations, the conversion of mycotoxins in the rumen fluid by microbes was considered as a first line of defense against toxic compounds present in the diet [23]. The metabolic process involved in the de-epoxydation of deoxynivalenol (DON) into de-epoxy DON (DOM-1) was characterized (Figure 2), and microbial degradation of mycotoxins was considered to be an effective way to decontaminate feed [24,25,26]. Effects of the rumen microbiota on other trichothecenes than DON have been described for nivalenol (NIV), diascetoxyscirpenol (DAS) and monoacetoxyscirpeneol (MAS), and T-2 and HT-2 toxins [18,19,27,28]. Together, bacterial and protozoal fractions are capable of T-2 toxin degradation, but the protozoal fraction seems to be the most active [27]. A recent study conducted in an in vitro rumen model at concentrations of mycotoxins that have no effect on the production of volatile fatty acids showed that pH and lactation can change the disappearance kinetics of DON and NIV [29].

While de-epoxydation of DON to DOM-1 by the microbiome of ruminant species is an efficient mechanism for the detoxification of DON, this metabolism appears to be less important in monogastric species. Indeed, although transformation of DON into DOM-1 by the pig microbiota has been characterized, degradation was poorly effective or occurred in the last portion of the intestine and excreta, i.e., of the fraction of the toxin that was not absorbed in the small intestine [25,30,31]. The main reason for the interest in the microbial degradation of mycotoxins in ruminants is the fact that the transformation occurs in the rumen, before absorption of mycotoxins in the small intestine (Figure 2). Consequently, ruminant species are among the most resistant animal species to DON, while pig is the most sensitive [4,6]. Incubation of 3-acetyldeoxynivalenol (3-ADON) with isolated human fecal samples in anaerobic conditions failed to reveal the formation of DOM-1, in contrast to in pigs, rats, mice, and chickens but which is also the case in dogs and horses [25,31,32,33]. Chicken intestinal microbes were able to completely convert non-acylated trichothecenes (4-deoxynivalenol, NIV, and verrucarol) into their de-epoxy metabolites [34]. In the same way, large amounts of de-epoxy metabolites were found for 15-MAS and de-epoxydation was the most important transformation for 4-acetylnivalenol, HT-2 toxin, and T-2 triol [34]. 

Deacetylation of trichothecenes is observed in both ruminant and in monogastric species, but the mechanisms involved in deacetylation are probably different (Figure 2). Incubation of 3ADON with isolated human fecal samples under anaerobic conditions revealed that a large amount of 3ADON was hydrolyzed into DON after 48 h of incubation [32]. Similar results were obtained with microbiome obtained from pig feces and ileal digesta [30], and an in vivo study in pigs confirmed deacetylation of 3ADON occurred probably both in the gut and the liver [35]. Studies conducted in mice, chickens, ducks, and pigs revealed faster and more efficient uptake of fusarenone X (FUS-X), compared to NIV, and rapid deacetylation of FUS-X into NIV after being absorbed, suggesting that the intestinal microbiota play no role in the metabolism of FUS-X [36,37,38]. Deacetylation of trichothecenes was the only transformation found for diacetylated trichothecenes (DAS and neosolaniol) and T2 toxin incubated with chicken intestinal microbes, and was the predominant pathway for monoacetyl trichothecenes (3ADON, 15ADON, and FUS-X) [34]. A recent investigation on human gut bacteria also revealed that *Prevotella copri* and *Butyrivibrio fibrisolvens* efficiently de-acetylated T-2 and DAS, at a concentration of mycotoxin that has no effect on bacterial growth [39].

**Figure 3:**
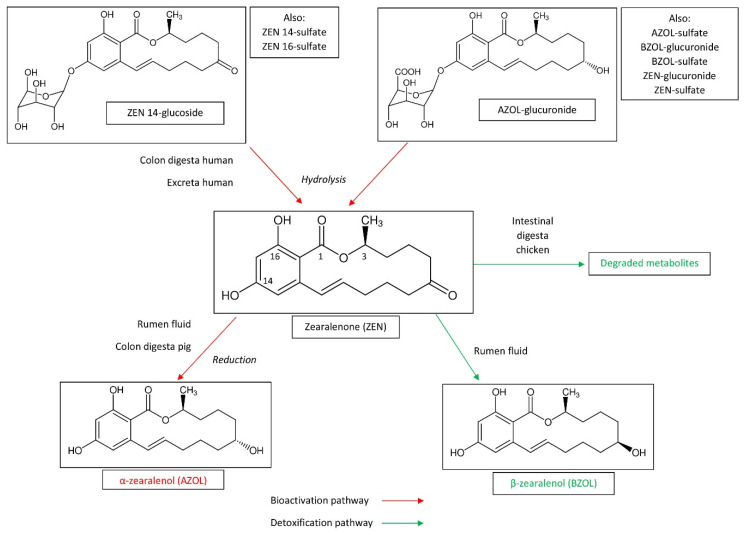
Biotransformation of zearalenone by the gut microbiota.

Not only trichothecenes can be metabolized in ruminant species [40]. Aflatoxin B1 (AFB1) can be transformed into aflatoxin M1 (AFM1) and aflatoxicol within the rumen, which could contribute to low bioavailability of AFB1 [19,27,41,42]. AFM1 and aflatoxicol have mutagenic properties and cannot be considered as detoxified forms. Ochratoxin A (OTA) was cleaved to ochratoxin α (OTα) and phenylalanine, which are detoxified compounds [19,43,44,45]. Interestingly, species differences were identified in the metabolism of AFB1 and OTA by the rumen fluid, but the production system also seems to influence this metabolism. The microbiota of high-production dairy and beef systems seem to be less effective in the degradation of AFB1 and OTA, probably because of ruminal acidosis, which has been demonstrated to reduce the ability of the rumen fluid to transform mycotoxins [46,47]. OTA metabolism by sheep microbiota also seem to be less effective than cattle microbiota, particularly when OTA is present in diets with a high proportion of concentrates [48]. As already mentioned for DON, OTA microbial degradation in non-ruminant species like pigs takes place in the large intestine and therefore occurs after having passed the major absorption site [49]. Consequently, even though microbial degradation in the large intestine may be efficient, it does not play a significant role in the animal’s health. Zearalenone (ZEN) is a xenoestrogenic mycotoxin whose metabolism, toxic effects, and neutralization strategies have been the subject of a recent review of the literature [50]. Transformation of ZEN by the gut microbiota is summarized in Figure 3. ZEN was reduced by ruminal microbial population to α-zearalenol (AZOL) and to a lesser degree to β-zearalenol (BZOL) [19]. Because AZOL was shown in an estrogenic bioassay to be 60-fold more potent than ZEN, this transformation can be considered as bioactivation of the toxin [51]. In pig, ZEN is metabolized in AZOL by the microbiota of the large intestine (particularly the colon), whereas microorganisms in the small intestine exhibited no transforming activity [31]. It should be noted that ruminant species are notably less sensitive to ZEN than pig, despite the fact ZEN was bioactivated into AZOL in the rumen [4,6,51]. Assays concerning the biodegradation of fumonisins revealed that fumonisin B1 (FB1) is poorly metabolized by the ruminal microflora while the cecal chime of pigs partially hydrolyzed the toxin [23,52,53,54]. Thus, transformation of FB1 by the gut microbiota is not the explanation for the tolerance of ruminant species to relative high levels of FB1 in feed, and late hydrolysis of FB1 is not sufficient to protect pigs from its toxicity [4,6]. The stability of PR toxin, mycophenolic acid, and roquefortine C were also measured in an in vitro rumen fermentation model [55]. Mycophenolic acid and roquefortine C were partially stable in this model whereas no PR toxin was recovered after 48 h of incubation. A recent study conducted in an in vitro rumen model at mycotoxin concentrations that have no effect on volatile fatty acid production showed that roquefortin C and enniatin B were partially stable, with the pH and stage of lactation having an effect on the disappearance kinetics of enniatin B but not of roquefortin C [29]. Gliotoxin was unstable in an in vitro rumen environment with 90% disappearance after 6 h of incubation [56]. A recent review of the literature on beauvericin and enniatins reveals that the bioavailability of these mycotoxins is generally reduced by the action of the gut microbiota and probiotics, but the mechanisms behind this reduction remain imperfectly known [16].

#### 2.1.2. Bioactivation of Masked Mycotoxins

Of major concern is the effect of gut microbiota on “masked mycotoxins”. The expression “masked mycotoxins” is used to characterize plant-derived mycotoxin metabolites, mainly glucose- and sulfate-conjugated forms, of DON, T2-toxin, and ZEN. The word masked is used to highlight the sequestration of these metabolites in the plant cell vacuole, which reduces their phytotoxicity [57,58]. By extension, “masked” is also often used for conjugated mycotoxins formed in animal tissues. Because of their modified form, masked mycotoxins are not detected when dosing the parent compound using conventional analytical techniques. Thus, the determination of the concentration of conjugated mycotoxins in food and feed requires the measurement of each conjugated form, or their hydrolysis, prior to measurement of the parent compound [57]. Because hydrolysis of aliments is common in the gut before nutriment absorption, the question arises whether such hydrolysis can occur on masked mycotoxins in the gastrointestinal tract [59]. In vitro studies conducted with DON-3glucoside revealed that hydrolysis in the upper gastro-intestinal tract prior to absorption of the toxin is unlikely [60,61,62,63], while human fecal microbiota was able to hydrolyze DON-3glucoside [62,64]. A recent investigation on the ability of 14 strains of human gut bacteria to break down masked mycotoxins at a concentration of mycotoxin that has no effect on bacterial growth revealed that *Butyrivibrio fibrisolvens*, *Roseburia intestinalis*, and *Eubacterium rectale* hydrolyze DON-3-β-glucoside, HT-2-β-glucoside, and NIV-3-β-glucoside while *Bifidobacterium adolescentis* and *Lactiplantibacillus plantarum* hydrolyze DON-3-β-glucoside only. None of the bacteria were capable of hydrolysis of α-glucosides [39]. Toxicokinetic studies in rats and pigs confirmed that that the majority of the DON-3glucoside was excreted as DON and DOM-1, but the oral bioavailability of DON-3glucoside and its metabolites seems to be lower than DON, suggesting that DON-3glucoside is of less toxicological relevance than DON [65,66,67]. Studies in pig also revealed that the microbiota obtained from the jejunum hydrolyzed DON-3glucoside very slowly, while the microbiota obtained from the ileum, cecum, colon, and feces hydrolyzed DON-3glucoside rapidly and efficiently [68]. By contrast, results in broiler chickens indicated that DON-3glucoside is not hydrolyzed into DON in vivo [67].

The study of the stability of the conjugated forms of ZEN is of particular interest as they can identify differences among mycotoxins and the forms in which they are conjugated. Glucosides and sulfates are formed in plants while glucuronides and sulfates are formed in animals. Some studies suggest that glucoside-conjugates could be more resistant to acid hydrolysis than sulfate-conjugates [69], whereas hydrolysis of ZEN-glucoside was demonstrated in other studies on swine [70]. In vivo studies demonstrated that ZEN was formed from ZEN-14glucoside in the stomach of rats, whereas DON-3glucoside was hard to hydrolyze [71]. In vitro studies conducted with ZEN-14glucoside and ZEN-14sulfate also revealed that human colon microbiota hydrolyze conjugated forms of ZEN, while it was unable to hydrolyze DON-3glucoside under the same conditions [62]. Interestingly, ZEN-16glucoside was also hydrolyzed by the human fecal microbiome [72]. Toxicokinetic studies conducted with different conjugated forms of ZEN confirm the intestinal hydrolysis of these compounds in pigs, without it being possible to say whether this hydrolysis is related to gastric acidity, the digestive enzymes of the pig, or an activity of the intestinal microbiota [73,74]. Intestinal digesta obtained from broiler was able to degrade ZEN into unknown metabolites that differ from AZOL, BZOL, zearalanone, α-zearalanol, and β-zearalanol [75]. A recent study confirmed that acid conditions were as potent as glucuronidases and sulfatases in the hydrolysis of the conjugated forms of AZOL and BZOL [76]. Finally, comparison of the stability of glucosides, sulfates, and glucuronides suggests that some conjugated forms are only hydrolyzed in the large intestine whereas others are hydrolyzed in the stomach or in the jejunum prior to absorption of the parent compound [62]. Although masked fumonisins, such as protein-bound fumonisins, and masked OTA, such as β-glucosides, have been described [57], little is known about the impact of the gut microbiota on the liberation of these forms.

#### 2.1.3. Microbial Degradation of Mycotoxin and Feed Additives

Observation of anaerobic de-epoxydation of DON into DOM1 in ruminant species opened the door to different defense strategies against mycotoxins that aimed to degrade the mycotoxin prior to its absorption in the gut, using purified microorganisms or enzymes added to the feed [18,19,20]. Very recent reviews of the microbial degradation of mycotoxins have been carried out [26,77,78], and here we focus on those related to the microbiota. Anaerobic DON-transforming microorganisms were isolated from bovine rumen content by screening the microbes in the rumen [79]. Culture of *Eubacterium* BBSH 797, a Gram-positive bacterial strain capable of DON degradation in vitro, was shown to be effective in counteracting the toxic effects of DON in broilers [80,81,82]. Good degradation rates of DON were also observed for various strains of microbes obtained from pig feces, and *Eggerthella* sp. DII-9 and *Slackia* sp. D-G6 strains that were isolated from the guts of chickens [26]. Epoxydase produced by *Eubacterium* BBSH 797 was identified as the key enzyme in the transformation of the epoxyde group of DON to DOM-1 [83]. Other biodegradation processes of DON involve oxidation of DON into 3-keto--DON or 3-epi-DON, acetylation, and glycosylation, but none of the microorganisms capable of these transformations are obtained from the gut microbiome [26,84,85,86]. A recent review of the origin of bacteria involved in the degradation of DON reported that around half originated from the gut microbiota [26]. Other Gram-positive bacterial strains used as mycotoxin-biotransforming agents belong to the genera *Bacillus*, *Nocardia*, *Corynebacterium*, *Mycobacteria*, *Rhodococcus*, and *Curtobacterium*. Gram-negative aerobic bacteria from the genera *Flavobacterium*, *Pseudomonas*, and *Alcaligenes,* but also fungi and yeasts, such as *Saccharomyces cerevisiae*, were shown to have the potential ability to biodegrade mycotoxins. Interestingly, some of these germs are also known to be pathogens in animals, consequently their safety should be carefully evaluated before being used as feed additives [83,84].

Biotransformation of ZEN by the normal gut microflora of pigs has been demonstrated in vitro, and AZOl, which is more estrogenic than ZEN, was formed during the process [31]. By contrast, the ANSB01G isolate, a *Bacillus subtilis* strain taken from normal broiler intestinal digesta, was shown to degrade ZEN into unknown metabolites that differ from AZOL, BZOL, zearalanone, α-zearalanol, and β-zearalanol [75]. Approximately 1000 colonies of *Bacillus* sp. were screened and four strains capable of degrading ZEN were isolated, among which *B. amyloliquefaciens* ZDS-1 displayed the greatest activity [87]. Testing the probiotic potential of *B. amyloliquefaciens* LN suggested that this strain could be used as a feed additive to reduce the concentrations of ZEN in feedstuffs [88]. Other strains of *Bacillus* tested include *B. licheniformis* CK1, *B. subtilis*, *B natto*, *B. cereus* BC7, and *B. velezensis* A2 [77]. Different strains were screened for their ZEN detoxification capability and their potential use in the feed industry [89]. An enzyme involved in the degradation of ZEN was isolated from a strain of *Clonostachys rosea*, and different applications in plant and feed were reviewed [77]. Interestingly, the enzyme was expressed in *Lactobacillus reuteri* Pg4, a probiotic strain in broilers [90]. However, further studies are necessary to determine the safety and efficiency of this probiotic before its use in animal feed. Other mycotoxins, such as patulin, gliotoxin, sterigmatocystin, and ergot alkaloids, can be efficiently biotransformed by enzymes that are present in the liver of humans and animals, or in different microbes present in the environment, but the impact of the gut microbiota on these process is not yet fully understood [17,86].

Microbial degradation of AFB1 has been investigated as the enzymes involved in this process [83,86]. Several studies on the effect of probiotics on AFB1 toxicity were conducted in animals. Because most of the beneficial effects of the use of probiotics in the course of feed contamination by aflatoxins appear to be linked to binding of the toxin to the cells walls, they are reviewed in Section 2.2 below. OTA is hydrolyzed by gut microbiota in OTα and phenylalanine, whose toxicity is considerably lower than the parent compound, and different commercial hydrolase enzymes have been screened for their potential use as feed additives [91]. Carboxypeptidases produced by *Bacillus amyloliquefaciens* ASAG1 and *Lysobacter* sp. CW239 were shown to biodegrade OTA [92,93]. *Trichosporon mycotoxinivorans* also cleaves OTA into *phenylalanine* and OTα in vitro and its inclusion in their diet blocked the detrimental effects of OTA on several variables of the immune system in broilers [94]. OTA degradation was also demonstrated in a colon reactor in the presence of gut microbiota, and a difference was observed between the ascending and the descending part of the colon [95]. However the biodegradation of OTA by the enzyme obtained from the gut microbiota has few applications in food and feed [84,86,96]. FB1 is hydrolyzed to form hydrolyzed (HFB1) and partially hydrolyzed FB1 whose toxicity is considered to be low compared with the parent compound [97]. Biotransformation occurs naturally in the gut of different animal species but only at a low level [98,99,100]. Carboxylesterase FUMD from *Sphingopyxis* sp. has been shown to be an effective enzyme in the hydrolysis of FB1 [98]. FUMD is efficient in the hydrolysis of FB1 in the gut of turkey, broiler, and swine, and provides partial protection against the toxic effect of FB1 on sphingolipid metabolism [101,102]. A combination of *Eubacterium* BBSH797 and carboxylesterase FumD was shown to efficiently neutralize the effects of diets mono- and co-contaminated with DON and fumonisins in pigs [103].

### 2.2. Adsorption of Mycotoxins

The role of the gut microbiome in the toxicokinetics of mycotoxins is not limited to biotransformation. Binding of mycotoxins leading to a decrease in their bioavailability has been demonstrated in vivo and in vitro, and fungi that are present in the rumen microbiota could play a role in this process [78,104]. In the rumen, AFB1 is at least partially sequestered by yeast cell wall-derived extracts [105,106]. Esterified glucomannan reduced AFM1 contamination of milk in late-lactation Holstein cows fed AFB1-contaminated feed [107]. However, the binding properties of AFB1 by organic materials, such as yeast products, are generally less than those observed with the clay minerals [108]. The mechanism of detoxification of aflatoxins based on native/standard probiotic bacterial strains was recently reviewed [78]. *Lactobacillus* strains are the most widely tested species, and around half of them come from avian and pig isolates of the gut [109]. Most of the detoxification due to the *Lactobacillus* strains seems to be related to binding of AFB1 and AFM1 [78]. In vitro detoxification of AFB1 by probiotic *Saccharomyces cerevisiae* yeast appears to be similar to that observed with *Lactobacillus* [109]. The mechanisms responsible for the binding properties of probiotics involve physical adhesion of aflatoxins to the carbohydrate components of the microbe cell wall by non-covalent interactions corresponding to the formation of van der Waals interaction, electrostatic interactions, and hydrogen bonds [78]. Fewer studies of the bacterial probiotics strains of the genus *Bacillus* and *Streptomyces* have been conducted for their effect on AFB1 than studies of strains of *Lactobacillus* and these strains also appear to be involved in the degradation of the aflatoxins [78]. Other Gram + bacteria belonging to the genera *Bifidobacteria*, *Rhodococcus*, *Cellulosimicrobium*, *Corynebacterium*, *Streptomyces*, and *Actinomycete*, and several Gram – bacteria belonging to the genera *Klebsiella*, *Pseudomonas*, *Enterobacter*, *Stenotrophomonas*, *Brevundimonas*, and *Mycobacterium*, but also strains of *Escherichia coli* and *Myxococcus fulvus*, are reported to be effective in aflatoxin detoxication. Fungal species, other than *S. cerevisiae*, reported to be effective in aflatoxin detoxication include *Candida utilis*, *Rhizopus* spp., *Aspergillus niger*, *Trichoderma* spp., *Phoma* spp., *Phanerochaete chrysosporium*, and *Pleurotus ostreatus* [78]. Even if all these microorganism are not used as probiotics they could be of interest in food processing, such as beer making, or in feed processing, such as silage fermentation [110,111].

Adsorption of mycotoxins to the cell walls has also been described for DON [26]. Like for aflatoxins, the genus *Lactobacillus* and *Saccharomyces cerevisiae* yeast are the most widely studied, followed by *Streptococcus* and *Enterococcus* [26,109]. Degradation can co-occur with binding and contribute to the overall reduction in DON content [26]. However, DON appears to be relatively resistant to the reduction in concentration when incubated with *Lactobacillus* and *S. cerevisiae* compared to what was observed in the case of AFB1 [109]. Comparison of the reduction in T-2 toxin concentration observed with 12 strains of *Lactobacillus* sp. and 6 strains of *S. cerevisiae* showed similar results for this toxin to the ones measured for AFB1, but no information was provided on the mechanism responsible for the decrease in concentration measured [109]. In the same study, a marked reduction in concentration was observed for FB1 and FB2 [109]. Because no bioactive metabolite of FB1 and FB2 has been described to date, the reduction in the observed concentration can be considered as beneficial, irrespective of whether the mechanism involved was due to toxin binding or to toxin hydrolysis [97].

The effects of microbes on ZEN vary with the genus/strain studied. While some *Bacillus* strains degrade ZEN into non-toxic metabolites, together, adsorption and metabolism could be important in the mechanism that reduces the concentration of ZEN when the toxin is incubated with *S. cerevisiae* and *Lactobacillus*. Indeed, it has been observed that ZEN binds to the β-D-glucans of *S. cerevisiae*, and together, the reticular organization of β-D-glucans and the ratio of β-(1,3) to β-(1,6), play a role in the efficacy of adsorption [112]. Other studies reported that elimination of ZEN from in vitro medium containing *S. cerevisiae* was mainly due to its biotransformation into BZOL and AZOL, whereas the adsorption of the toxin seems to be low [113]. A recent study demonstrated that a *Bifidobacterium* sp. was able to neutralize ZEN in an homogeneous process, with about 88% of ZEA biosorption [114]. Thus, studies conducted with ZEN underline the importance of understanding the mechanism involved in the reduction in toxin concentration measured in vitro when mycotoxins are incubated with probiotics [115,116]. A decrease in concentration due to the fixation of the mycotoxin will only be effective in vivo if fixation survives intestinal absorption. A decrease in concentration due to the formation of AZOL cannot be considered as detoxification, but on the contrary, as bioactivation, as AZOL is 60 times more estrogenic than ZEN [51]. These works also underlined the interest of using enzymes and purified fractions of microbes, rather than whole organisms. In addition to increased safety linked to the control of the mechanism involved in detoxification, decontamination by purified enzymes or cells wall is expected to be more substrate specific, without affecting the texture of the food or causing loss of nutrients [77].

## 3. Effects of Mycotoxins on the Gut Microbiota

The effects of mycotoxins on the gut microbiota correspond to changes in the gut microbiota population. These changes can occur at the phylum, the genus, or the species level. These changes can be the direct consequence of antimicrobial properties of the mycotoxins or can be secondary to the toxic effect of mycotoxins on the gut cells and the leakage of antimicrobial compounds. Because the concentration of mycotoxins in the different parts of the gut varies considerably due to absorption and biliary excretion, but also due to the effect of the microbiota on mycotoxins, and because the microbiome varies in composition depending on which part of the gut is being analyzed, the effects of mycotoxins on the gut microbiota are quite difficult to characterize and results may vary with the experimental design of the study [117,118].

### 3.1. Antimicrobial Properties of Mycotoxins

Because of their fungal origin, the antimicrobial properties of mycotoxins were hypothesized as soon as these compounds were purified, and first in vitro studies with AFB1 confirmed that the toxin can inhibit bacterial growth [119]. Patulin, citrinin, ergot alkaloids, and other mycotoxins were screened for their activities against Gram+ and Gram− bacteria, but their use as an antibiotic has been discouraged due to their toxicity [120]. Similarly, a recent review of the bibliography on beauvericin and enniatins revealed that these mycotoxins have antibacterial properties at doses close to cytotoxic doses [16]. Different in vitro studies were conducted with rumen fluid to measure the effects of mycotoxins on the microbiota. Because the microbiota present in the rumen are actively involved in the degradation of raw material prior to its absorption, most of the effects of mycotoxins were indirectly characterized as their effects on digestibility. Studies with AFB1 revealed that the toxin reduced dry matter digestibility, gas production, and concentrations of ammonia-N in an anaerobic batch culture system with ruminal microbial population collected from steers [121]. Studies with ovine rumen fluid showed that microbial activity was partially inhibited by AFB1 as determined by the inhibition of digestion of alfalfa hay [27]. In other studies, the addition of AFB1 to feed with high concentrations in alfalfa hay or ryegrass hay reduced gas production and the ammonia-N concentrations but did not reduce dry matter digestibility or volatile fatty acid patterns [122]. It should be noted that all these results were observed at a concentration of AFB1 between 0.3 and 1 mg/L, while a concentration of 9 µg/L appeared to have no effect on these variables [123]. In the same way, most of the mycotoxins present in silage were shown to cause alterations in the microbial digestion of dry matter and the production of microbial end products [55,56,124]. Consequently, patulin, glyotoxin, mycophenolic acid, and roquefortine C were suspected to have an impact on health when present at high concentrations in silage fed to ruminant species [55,56,124]. Because some mycotoxins produced by *Monascus* spp. were shown to be capable of reducing methanogenesis without affecting fermentation and feed digestion, it is hypothesized that these toxins could reduce the environmental impact of ruminant production, but this hypothesis needs to be confirmed in farm conditions [125].

DON failed to demonstrate antimicrobial activity against *Ruminococcus albus* and *Methanobrevibacter ruminantium* at concentrations of up to 100 mg/L while the growth of both organisms was inhibited by fusaric acid at 15 mg/L [126]. Another study in an in vitro rumen fermentation model showed that gas production and the concentrations of ammonia-N and volatile fatty acids were reduced by DON at a concentration of 40 mg/L [127]. By contrast, the fermentation of organic matter, crude protein, and neutral detergent fiber were increased in a fermentation model using a contaminated diet containing 64.9 mg DON/kg. The authors hypothesized that nutrient availability was enhanced because of modifications to the plant cell wall due to fungal development [128]. DON at a concentration of 5 mg/kg in the diet had no effect on the fermentation of organic matter nor on microbial protein synthesis. However, a change in the microbial community composition of the genus *Clostridium* was noted while no effect was observed on the genera *Archaea*, *Fibrobacter*, *Bifidobacterium*, *Bacillus*, and *Bacteria*. The authors hypothesized that the observed changes in the *Chlostridium* population could reduce cellulolytic activity of the rumen microbiota [129]. Interestingly, the same authors highlighted the marked differences in the potential extent of organic matter fermentation and crude protein degradation depending on the rate of concentrate in the diet, which could have a bigger impact on the microbiota present in the rumen than the mycotoxins [129]. FB1 at concentrations of up to 100 mg/kg in the diet had no effect on the microbial efficiency measured in a ruminal microbial population [54]. Studies using an in vitro model of colon fermentation in humans reported that OTA has only a weak effect on fermentation but could induce some minor changes in the *Bifidobacterium* population [95]. Taken together, these works suggest that the antimicrobial properties of mycotoxins on gut microbiota are only observed at relatively high concentrations of mycotoxins in the digesta.

### 3.2. Cell Toxicity and Leakage of Antimicrobial Products (AMPs)

Because of its position in the sequence of mycotoxin intake, the gut is the first organ on which mycotoxins can exert their toxicity. Four anatomic/histologic layers can be distinguished from the lumen of the gut to the peritoneal cavity: the mucosa, the submucosa, the muscularis propria, and the serosa. The mucosa is composed of three layers: the epithelium, the lamina propria, and the muscular mucosae (Figure 1). The epithelium layer contains the epithelial cells, which are enterocytes and the goblet cells in the villi, and the enterocytes and the Paneth cells in the crypts. Epithelial cells are interconnected by desmosomes, tight junctions, and adherens junctions to form a mechanical linkage of adjacent cells, thereby creating a physical barrier that blocks the entry of pathogens and large particles but remains permeable to dietary nutrients, electrolytes, and water. This barrier is completed by a chemical barrier composed of mucus, cytokines, and antimicrobial peptides secreted by the epithelial cells. The lamina propria contains capillary and gut-associated lymphoid tissue (GALT) that is formed by different cells of the immune system, including lymphoid cells, macrophages, and dendritic cells, and compose the immunological barrier. Secreted immunoglobulin A and cytokines are produced by cells of the immune system to complete the barrier produced by the epithelial cells. Because the microorganisms are living in the close environment of epithelial cells, it was hypothesized that changes in cell secretions and in cell receptors could change the gut microbiome [14,130]. Recent reviews of the literature on the impact of mycotoxins on gut are available, so here we simply summarize which mechanisms behind gut toxicity secondary to mycotoxin exposure can alter the gut microbiota [15,16,131,132].

The toxicity of mycotoxins to the oral mucosa of most necrosing compounds, including T2 toxin and stachybotrys toxin, has been known for many years [133,134]. Consequently, experimental models to assess the toxicity of mycotoxins have been developed, the first of which used epithelial cells lines. Among them, the human Caco-2 cell line, and the porcine intestinal epithelial cell lines IPEC-1 and IPEC-J2, became reference models to investigate barrier function and cell toxicity. Freshly isolated intestinal primary cells from various animal species are also used as 3-D cell culture models and intestinal explant models [15,16,132]. The main limitation of these models may be the lack of bioactivation of mycotoxins that could occur in the liver but also in the gut due to microbial transformation, as reviewed in Section 2.1.2 above. Nevertheless, several studies on mycotoxins toxicity were performed on these models, including the evaluation of non-specific end points, such as necrosis and apoptosis, but also more specific end points of target organ toxicity, such as the measurement of transepithelial electrical resistance, the determination of the paracellular flux of marker substances across the cell monolayer, and the assessment of the expression of thin junction proteins. Recent reviews of the literature summarized the toxic effects of mycotoxin, mycotoxin by mycotoxin, model by model [15,16,132]. These studies concluded that nearly all the mycotoxins assayed inhibited cell viability and led to cell cycle arrest and apoptosis or necrosis, but the doses assayed were sometimes high. The mechanisms involved in toxicity included induction of mitochondrial reactive oxygen species and Ca2+-mediated myosin light-chain kinase activation, inhibition of claudin-1 expression, and decreased mitogen-activated protein kinase activation. The functional consequences of these alterations were increased intestinal permeability, and disruption of microvilli and thin junction proteins. These results strengthened studies conducted in vivo that demonstrated adverse effects on intestinal morphology in different animal species [15,16,132].

Not only epithelial cells are sensitive to mycotoxins; so are the cells of the immune system present in the GALT [15,16,132]. The immunomodulatory properties of mycotoxins have been described for years and have also already been reviewed [135]. However, an even greater number of studies demonstrated that aflatoxins, OTA, DON, fumonisins, and others are responsible for alteration of the immune response, but the lack of standard testing in immunotoxicity assays means the endpoints are difficult to establish. The effects of mycotoxins on the GALT can be due to the direct toxicity of mycotoxins, as described for enterocytes, but can also be the consequence of toxicity in the thymus, the spleen, and the bone marrow in which the cells of the immune system mature and grow [136,137,138,139]. 

Thus, the main consequences of mycotoxin toxicity in the cells present in the gut for the microbiota are changes in mucus secretion and increased production of cytokines, notably TNF-α and IL-1β, and an increase in secreted immunoglobulins A and antimicrobial peptides (AMPs), which alter the composition of the chime. Among the compounds secreted, more than 100 display antimicrobial activity and reviews of the literature on AMPs and their role in the gut microbiota homeostasis as the role of mucosal immunoglobulins in pathogen control and microbiota homeostasis are available [140,141,142]. When the concentrations of AMPs needed to observe antimicrobial effects are compared with the concentrations of mycotoxins at which antimicrobial activity was described and the concentrations of mycotoxins at which cytotoxicity occurred, it seems reasonable to assume that most of the changes in the gut microbiota secondary to exposure to mycotoxins are more likely to be secondary to the cytotoxicity of the mycotoxins than to their antimicrobial properties.

### 3.3. Changes in the Gut Microbiota Secondary to Mycotoxin Exposure

Changes in the gut microbiota secondary to mycotoxin exposure are listed in Table 1. These changes were reported in animal species as the gut microbiome and mycotoxin exposure vary strongly with the animal species. The experimental design of the studies reviewed also sometimes differed considerably, as did the dose and the duration of mycotoxin exposure and the methods used to characterize their effects on the gut microbiota. PCR-amplified 16S ribosomal DNA fragments became the most widely used method, while the interest of culturing microbes has considerably decreased due to their low sensitivity and specificity. Indeed, most of the microbes present in the gut will not grow in culture, and the culture of microbes generally does not enable analysis of microbiota at the phylum and genus levels. However, one study conducted with OTA showed that the combination of metagenomic and culture-based methods can be a valuable strategy to study mycotoxin and gut microbiome interactions, especially when the aim is to obtain bacterial strains of probiotic interest [143].

Several results showing the impact of mycotoxins on gut microbiota were obtained using feces that are easy to collect, but these results are not always representative of effects that occur on the different segments of the gut [8]. The gut microbiota is determined by genetics and environmental factors, and so there are notable differences in bacterial composition between species. For example, the phyla *Bacteriodetes* and *Firmicutes* are well established in rodents, pigs, and humans, while *Clostridiales* vary strongly [9,10,11,12,13,151,173]. Due to absorption and biotransformation, both the concentration and the form in which the mycotoxins are present in the different parts of the gut vary. Consequently, it is not surprising that the effects of mycotoxins on the gut microbiome vary depending on the portion of the gut analyzed, as observed for DON and ZEN in pig [157,161]. Surprisingly, similar effects of mycotoxins on the gut microbiota are observed although these vary markedly in their other effects on health [149,157,160]. This can be explained by the fact that most of the mechanisms involved in the effects of mycotoxins on the microbiota are due to toxicity occurring in the gut and not to their antimicrobial properties (see Section 3.1 and Section 3.2).

Another factor of variation in the studies reviewed in Table 1 is the time at which the results are observed not only with respect to the length of exposure to the toxin but also the age of the animal. Indeed, changes in microbiota composition have been reported with age but also with the composition of the feed, which varies with the age of the animals in farm conditions [174,175]. Most of the studies were conducted to measure the effect of a mycotoxin after a fixed time of exposure, but several suggest the effect of mycotoxins on microbiota varies with time [146,150,156,159,163,166,167]. Some studies reported transient effects that are partially or totally reversible [162,163,164,166,167]. The dose of mycotoxin is of course a determining factor in the results. Although most studies show that the effects on the microbiota follow a classical dose–response pattern (monotone curves in which an increased effect corresponding to the increase in the dose), this is not always the case, and no monotone dose–response effects were observed in some works [149,157,160]. This last observation suggests that the effect of mycotoxins on gut microbiota can alter the microbiota composition, which in turn, can modify the amount of mycotoxin transformed or bound, which can secondarily affect the impact of mycotoxin toxicity on the gut. Indeed, *Bacteroides* and *Lactobacillus* are commonly affected by mycotoxins (Table 1), and these species were reported to have strong detoxication properties. Interestingly, the concentration of DOM1 in feces was seen to increase with the duration of exposure in rat, suggesting the microbiota increased its rate of de-epoxydation with time [147]. However, in that study, only slight changes were observed in the microbiota community with time except for an increase in the relative abundance of the *Coprococcus* genus, but the ability of this genus to de-epoxy DON into DOM1 has not previously been reported. A last observation is worth making regarding the doses of mycotoxins used. Even though most of the studies were conducted using a relatively high level of mycotoxins in feed, some conducted using low levels suggest that mycotoxins may have an effect on the microbiota at doses that are not reported to be cytotoxic [152]. Finally, although the effects of mycotoxins on gut microbiota are sometimes difficult to compare, there is a scientific consensus on the importance of these effects, and well-controlled study designs are essential to ensure the studies are repeatable and produce consistent results.

## 4. Combined Effects

The dichotomy in studying mycotoxins and gut–microbiota interactions between the “effect of mycotoxins on the gut-microbiota” and the “effect of the gut-microbiota on mycotoxins” enables a better understanding of the mechanisms involved in these interactions, but the impact of these interactions on health is the result of the combination of the two phenomena. If we exclude the direct transformation or binding of mycotoxins by the gut microbiota that occur before changes in the gut microbiota become visible, the impacts the interactions between mycotoxins and gut microbiota have on heath are more particularly studied in terms of the capacity of the organism to defend itself against gut pathogens. Other major consequences of these interactions for health are suspected in carcinogenesis and chronic diseases in human.

### 4.1. Alteration of the Capacity of Defense against Pathogens

Several pathogens can develop in the gut, and some can cross the gut barrier and invade the body. Defense against pathogens, i.e., the gut barrier effect, is the result of the synergy of three complementary mechanisms/barriers: (1) a microbiota barrier formed by non-pathogenic microbes that colonize the gut; (2) a physical-chemical barrier formed by the epithelial cells and their secretions; and (3) an immune barrier formed by the GALT (Figure 1). Alterations to the gut barrier function lead to mucosal infection or translocation of bacteria and their products, namely pathogen-associated molecular patterns, to the whole body. Bacterial and chemical translocation is a normal phenomenon when it occurs at a low rate as it is responsible for the development of immune tolerance to commensal flora and food, and for antigenic exposure to the immune system that prepares the organism for invasion by pathogens [176]. Several reviews of the literature highlight the importance of food components and dietary habits for gut microbiota composition and healthy barrier function [176,177,178]. The effects of mycotoxins on mucosal microbial infection and related pathogenesis have also been reviewed [179]. Because the onset of mucosal infection, with or without germ translocation, is closely linked to the pathogenicity of the germ studied, it is more likely to be investigated pathogen by pathogen [180,181]. 

Only a few studies are available on the effect of mycotoxin on enteric infection due to viruses, and those that are available are limited to reoviruses. Reoviruses infect the intestine following oral inoculation and infections are commonly non-pathogenic in adults. Reovirus infection is suspected to disrupt the host immune response to food antigen and contribute to the development of celiac disease and loss of oral tolerance to food [182]. Relative high doses of DON (10–25 mg/kg BW) increased reovirus L2 RNA excretion in feces while 2 mg/kg BW potentiated L2 RNA levels in Peyer’s patches [183]. These effects were accompanied by an increase in reovirus-specific IgA levels in feces and serum and increased specific IgA responses in lamina propria and fragment culture of Peyer’s patches. Suppressed Th1 and enhanced Th2 cytokine expression were also observed [183]. Additionally, a high dose of T2-toxin (1.75 mg/kg BW) by intraperitoneal injection suggested that T-2 toxin increased both the extent of GI tract reovirus infection and fecal shedding, which corresponded to both suppressed immunoglobulin and IFN-γ responses [184]. Even though these studies highlighted immunomodulatory properties of trichothecenes upon exposure to high doses, it is difficult to conclude that alterations in the microbiota barrier have an impact on reovirus infection because of the limited number of assays and because of the dose of mycotoxins used. 

The effects of mycotoxins were investigated in *Clostridium perfringens*, an anaerobic Gram-positive spore-forming bacillus that is a component of the gut microbiota in several animal species. Proliferation of *C. perfringens* is responsible for intestinal infections that manifest as enteritis, enterocolitis, or enterotoxemia depending on the virulence of the strain and its ability to produce toxins [185]. DON fed at a dose of 3–4 mg/kg in broilers increased the percentage of birds with subclinical necrotic enteritis due to *C. perfringens*. This effect was accompanied by a reduction of duodenal villus height and in the transepithelial electrical resistance, while DON had no effect on in vitro growth of the germ or α-toxin production and netB toxin transcription [186]. In another study, DON at a dose of 2.5–10 mg/kg feed for 35 days reduced the relative abundance of the *Clostridiaceae* genus and that of *Clostridium* whereas it increased the relative abundance of the *Clostridiales* genus in cecal digesta in broilers [169]. Other studies in pig suggested that DON at 8 mg/kg feed and ZEN at 0.8 mg/kg feed reduced the level of *C. perfringens* in the colon digesta [160]. AFB1 fed at 40 μg/kg of feed to broilers challenged with *C. perfringens* increased gut and liver lesions and reduced the production of IgA but had no effect on *C. perfringens* in excreta [165]. A study conducted using one-day-old broiler chicks fed 18.6 mg FB1+FB2/kg feed 15 days prior to a *C. perfringens* challenge showed that the birds fed FB developed a higher percentage of subclinical necrotic enteritis than the control group [170]. Histopathological analysis of the gut also revealed that villus height and crypt depth of the ileum were reduced by FB prior to infection, while the abundance of *Candidatus* Savagella and *Lactobacillus* spp. in the ileal digesta was decreased and the abundance of total *C. perfringens* was increased. Taken together, these results suggest that the effect of the mycotoxin on *C. perfringens* infection at the doses of exposure used were linked to alterations in the mucosal barrier and in the immunological barrier while the toxin has no antimicrobial properties in vitro. Changes were observed in the relative abundance of *Clostridium* in the microbiota, but it is not clear whether these changes were the cause or the consequence of enteritis. Additionally, while some studies showed that mycotoxins promoted enteritis, others reported that mycotoxins had no effect or even reduced the amount of *C. perfringens* present in the microbiota [170].

The interest of using *E. coli* to study the effects of mycotoxins on bacterial translocation is linked to the high variability in the pathogenicity of this germ. *E. coli* is both a common commensal Gram-negative bacterium of the gastrointestinal tract and one of the most important pathogens responsible for bloodstream, urinary tract, and intestinal infections [187]. The diarrhoeagenic strains are classified in sub-pathotypes that differ in their clinical manifestation and pathogenesis. Four pathotypes are enterohemorrhagic, enterotoxigenic, enteropathogenic, and enteroaggregative [187]. Aflatoxin (1–3 µg/kg) and fumonisins (50–350 µg/kg) were detected in the hemorrhaged mucosa of mature cattle in case of Shiga toxin-producing *E. coli* infection of the gut. In vitro culture suggested that the mycotoxins increased enterotoxin and pore-forming toxin activity [188]. A relatively high dose of DON (10 mg/kg BW) increased DNA damage in the intestinal epithelial cells of newborn rats whose gut was colonized by *E. coli* strains producing colibactin, without altering the composition of the gut microbiota [148]. Fourteen days of receiving OTA at a dose of 2 mg/kg increased histopathological damage to the kidneys and liver and increased atrophy of lymphoid organs of broiler chickens challenged with *E. coli* O78, compared to chickens fed OTA alone or chickens infected with *E. coli* alone [189]. FB1 at 200 mg/kg feed in turkeys inoculated with *E. coli* increased the number of colonies observed in the blood and tissue homogenates [190]. At a dose of 0.5 mg/kg BW in piglets, FB1 increased colonization of both the small and large intestines by the orally inoculated pathogenic *E. coli* ExPEC strain [191]. Further studies using 1 mg FB1/kg BW revealed a longer period of shedding by the F4^+^ ETEC *E. coli* strain after infection and a lower induction of the antigen-specific immune response after oral immunization. These changes were accompanied by reduced intestinal expression of IL-12, impaired function of intestinal antigen-presenting cells, reduced upregulation of major histocompatibility complex class II molecule, and reduced T cell stimulatory capacity [192]. Studies of the consequences of feeding mycotoxins to animals challenged with *E. coli* thus revealed increased damage not only to the gut but also to the liver and kidneys caused by exposure to mycotoxins because of bacterial or toxin translocation. The underlying mechanisms suggest that mycotoxins can both promote the pathogenesis of some strains and reduce the ability of the host to defend itself against the bacteria. However, it is not clear whether alteration of the microbiota barrier played a determining role in these effects as contradictory results were obtained on the effect of mycotoxin on the abundance of *E. coli* in the gut microbiota (Table 1).

*Salmonella* are of particular interest for evaluation of the risk of bacterial translocation because this Gram-negative pathogen is highly invasive and able to colonize the whole organism starting from the gut. Other benefits of studies conducted with *Salmonella* are the role of innate immunity in defense against this pathogen, and the existence of strains of mice that are resistant to *Salmonella* [193]. AFB1 fed at a dose of 470 µg/kg to broilers challenged with *Salmonella* Enteritidis adversely affected the birds’ intestinal barrier function, resulting in increased gut permeability but with no significant impact on the invasive potential of the bacteria [194]. DON fed at a dose of 2 mg/kg to male Balb/c mice (susceptible strain) challenged with *Salmonella* Enteritidis increased the onset of infection in the mesenteric lymph nodes and liver and increased *Salmonella* counts in the spleen [195]. In a porcine intestinal ileal loop model, 1 mg DON/L was shown to increase the invasion and translocation of *Salmonella* Typhimurium with a subsequent potentiation of the inflammatory response in the gut [196]. A high dose of T-2 toxin (1 mg/kg BW) increased mortality in *Salmonella*-resistant C3H/HeN mice challenged with *Salmonella* Typhimurium without affecting intestinal infection but increased the number of bacteria in the spleen [197]. Infection was accompanied by increased bacterial-related lesions in the spleen, kidney, and liver [198]. The conclusions of these works were that T-2 toxin led to immunosuppression, thereby promoting the infection. High doses of T-2 toxin in chickens challenged with *Salmonella* administered by intra-peritoneal injection also caused immunosuppression [199]. Other studies in chicken suggest that resistance to cecal *Salmonella* colonization may be reduced in animals fed high doses of T-2 toxin [200]. OTA fed at a dose of 3 mg/kg feed also increased the number of *Salmonella* in the duodenal and cecal contents of chickens orally challenged with *Salmonella* Typhimurium [201]. A high level of FB1 (150 mg/kg feed) administered to Japanese quail prior to *Salmonella* Gallinarum challenge increased clinical signs of diarrhea with bloody discharge and mortality [202]. By contrast, fumonisins fed to pigs at a concentration of 11.8 mg FB1+FB2/kg feed prior to a *Salmonella* spp. challenge led to inhibition of the ability of specific *Salmonella* lymphocytes to proliferate following exposure to a specific Salmonella antigen. However, the ingestion of fumonisins had no impact on *Salmonella* translocation or seroconversion in inoculated pigs [163]. Thus, most of the interactions between mycotoxins and *Salmonella* were investigated at relatively high doses of mycotoxins to demonstrate the immunomodulatory properties of the toxins. Several works conducted in vitro highlighted the role of macrophages in the defense against *Salmonella* and of the immunomodulatory properties of mycotoxins in macrophage and lymphocyte functions [15,16,196,203]. Finally, only a few studies suggest that, at a level of exposure below or equal to the regulatory limit or maxima recommended in feed, mycotoxins can have an impact on bacterial translocation. Alterations in the epithelium barrier and in the immunological barrier appeared to be the key mechanisms by which mycotoxins alter the gut barrier [15,16,132]. 

### 4.2. Other Effects on Heath

**Figure 4:**
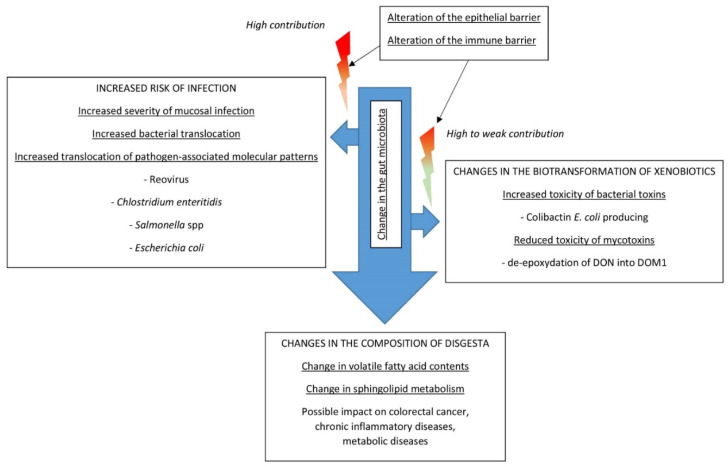
Impacts on health of changes in gut microbiota secondary to mycotoxin exposure.

The role of gut microbiota in the onset of chronic inflammatory diseases has long been suspected, and reviews of the literature on this topic are regularly updated [204,205,206,207]. The effects of interactions on health have been demonstrated or are suspected in colorectal cancer, irritable bowel disease, type 2 diabetes, non-alcoholic liver disease, cardio-metabolic diseases, mental or psychological diseases, autoimmune diseases, and malnutrition [204,205,206,207,208]. However, very little is known about the impacts that interactions between mycotoxins and gut microbiota may have on these diseases, and mycotoxins are not cited as known causes in dysbiosis in most reviews [204,205,206,207,208]. Proof of concept of the importance of changes in the gut microbiome related to mycotoxin exposure for health are provided by only a few studies and these are often conducted using relatively high levels of contamination. These works highlight the fact that alteration of gut microbiota secondary to mycotoxin exposure can lead to a range of alterations that are summarized in Figure 4. These effects are in addition to those of mycotoxins on intestinal cells and on the immune system, which were the subject of a very recent review of the literature, and which contribute greatly to these phenomena [12].

The diet and fecal microbiome appear to play a key role in the genotoxicity of xenobiotics and risk of colorectal cancer [208,209,210]. A study conducted in newborn rats whose gut was colonized by *E. coli* strains producing colibactin showed that DON administered at a dose of 10 mg/kg BW increased the phosphorylation of histone H2AX in the intestinal epithelial cells of the gut. An in vitro study demonstrated that the exacerbation of the genotoxicity of the *E. coli*-producing colibactin was time and dose dependent [148]. The authors suggest that the inflammation induced by DON could create an environment that allows colibactin to express its genotoxic potential [148]. This result is of particular interest because DON is not recognized to be carcinogenic and is classified in group 3 as “not classifiable as to its carcinogenicity to humans” by the international agency for research on cancer [211]. Recent studies highlighted the role of dysbiosis, and the presence of certain strains of *Escherichia coli*, among others, in patients suffering from colorectal cancer [208]. As previously discussed in Section 2, microbiota can alter mycotoxin toxicity. It was observed in rats fed 60 and 120 μg DON/kg BW for 40 days that the concentration of DOM1 in feces increased with the length of exposure, suggesting that the microbiota increased its rate of de-epoxydation of DON with time [147]. This result is of major interest because it was observed at a low dose, i.e., below the no-observed adverse effect level of DON estimated at 150 μg/kg BW/day in rat.

Pathogen-associated molecular patterns, including lipopolysaccharides, and metabolites of gut bacteria, such as short-chain fatty acids and sphingolipids, can activate different signaling pathways implicated in several biological functions [212]. A study conducted with OTA administered to ducks at a dose of 235 μg/kg BW showed that the toxin increased the relative abundance of lipopolysaccharide-producing *Bacteroides*, which led to inflammation in the liver. Antibiotic treatment of the ducks protected them against alterations in the liver, while transplantation of the intestinal microbiota obtained from OTA-treated ducks promoted liver inflammation in recipient ducks [172]. This work revealed that a change in the microbiota composition secondary to OTA exposure can increase blood lipopolysaccharides in the absence of infection, leading to chronic inflammation, which is also involved in chronic inflammatory diseases [213]. 

Increased cecal short-chain fatty acid concentrations and decreased glycoprotein and amino acid metabolism were reported in chickens fed 2.5 to 10 mg DON/kg feed [169]. This change was accompanied by a change in microbiota composition (Table 1). This result is of particular interest because short-chain fatty acid metabolites are suspected to play a key role in microbiota–gut–brain crosstalk and in chronic inflammation [214,215]. Interestingly, *Lactobacillus rhamnosus* GG supplementation supplied to mice receiving 1 to 5 mg DON/kg BW for 14 days increased the abundance of *Bacteroidetes* and the levels of the butyrate-producing genes to promote butyrate production [150]. Additionally, DON fed to pigs at a dose of 2.89 mg/kg feed led to several changes in the cecal microbiota and notably reduced the relative abundance of unclassified f_Lachnospiraceae that was positively correlated with average daily feed intake [158]. The authors suggested that microbial changes secondary to DON exposure could disturb appetite-regulating hormones and somatotropic-axis-hormone secretion, and that this mechanism could be a valid explanation for DON-induced anorexia [158].

Another alteration of health secondary to the interactions between mycotoxins and gut microbiota could be the consequence of the impairment of sphingolipid metabolism. Indeed, alteration of sphingolipid pathways is of particular concern given the role of these compounds in inflammatory diseases [216], and a recent study demonstrated that sphingolipids produced by gut bacteria enter the host and impact sphingolipid pathways in the host [217]. Sphingolipids are produced by gut bacteria, notably those of the phylum *Bacteroidetes*, including the common genus *Bacteroides*, whose abundance can change during exposure to mycotoxins (Table 1). DON administered to mice increased the relative abundance of *Bacteroidetes* in cecal digesta when administered at a dose of 5 mg/kg BW for 14 days but reduced the relative abundance of *Bacteroidetes* in excreta when administered at a dose of 10 μg/kg BW for 280 days [151,152].

## 5. Conclusions

The interactions between mycotoxins and gut microbiota were revealed very early on. Some of the differences in sensitivity in different animal species were thus explained by a protective effect of the microbiota against mycotoxin toxicity. This effect was associated with the degradation of the molecules into less toxic metabolites and a reduction in digestive absorption of mycotoxins. The characterization of the microbes involved in these reactions enabled the development of probiotics, and some of the currently sold probiotics derive directly from animal digestive flora. In parallel to this beneficial effect of the microbiota on the toxicokinetics of mycotoxins, a negative effect was recently demonstrated in the form of the hydrolysis of conjugated/masked mycotoxins. This hydrolysis, also related to gastric acidity and digestive enzymes, led to the release of mycotoxins in the digestive tract, which, added to the non-conjugated forms, contributed to the overall toxicity of contaminated food and feed. However, the interactions between mycotoxins and intestinal microbiota are not limited to effects of the microbiota on mycotoxins, and an increasing number of studies are characterizing effects of mycotoxins on the microbiota. The first of these works mainly focused on the disruption of the barrier effect provided by the gut and on the risk of bacterial translocation. However, the gut barrier results from synergy between three complementary mechanisms/barriers: (1) the barrier linked to the microbiota that colonizes the intestine; (2) the physical-chemical barrier formed by epithelial cells and their secretions; and (3) the immune barrier. Studying infection during exposure to mycotoxins revealed that the risk of bacterial translocation resulted mainly from the toxicity of mycotoxins towards intestinal cells and the immune system, at least at high doses. More recently, a growing number of studies have shown that mycotoxins can disturb the gut microbiota by modifying the relative abundance at the phylum, genus, and species level. This effect led to the design of new approaches to study the impacts of mycotoxins on health. Disturbance of the gut microbiota secondary to mycotoxin exposure is now suspected to be involved in the genotoxicity of xenobiotics and in the occurrence of several chronic diseases in humans, notably through the modification of the volatile fatty acids and sphingolipid contents of the digesta. These new mechanisms of toxicity are in addition to the cytotoxic and the proinflammatory effects of mycotoxins already observed on the intestinal mucosa and on the immune cells. Although the studies on the interactions between gut microbiota and mycotoxins occurring at low doses are still in their infancy, and much work is needed before we can conclude on the real impact these interactions have on health, these approaches open the way for new scientific and risk assessment challenges.

## Figures and Tables

**Table 1:** Effect of mycotoxins on gut microbiota composition.

Species	Exposure *	Method of Aanalyze	Sample Analyzed	Result	Reference
Rat	AFB1: 5, 25, 75 μg/kg BW 28 days	16S rRNA gene sequencing	excreta	Decreased phylogenic diversity No consistent pattern of increase or decrease at phylum level	[144]
Rat	AFB1: 25 μg/kg BW 28 days	16S rRNA gene sequencing	excreta	*Firmicutes* (82%), *Bacteroidetes* (13.5%) the most abundant *Proteobacteria* (3.3%) *Actinobacteria* (1.7%) and *Saccharibacteria* (1%) No effect on microbiota richness Increased abundance of *Alloprevotella* spp decrease in *Prevotella_9*.	[145]
Rat	DON: 100 μg/kg BW 28 days	RT-PCR	excreta rats inoculated with human fecal flora	Variation of microbiota composition with time Increased concentration of *Bacteroides* and *Prevotella* genera on day 10–20 Reduced expression of *Escherichia coli* on day 27	[146]
Rat	DON: 60, 120 μg/kg BW 40 days	16S rRNA gene sequencing	cecal digesta	*Firmicutes* and *Bacteroidetes* were the most abundant Increase in the relative abundance of *Coprococcus* genus	[147]
Rat	DON: 2, 10 mg/kg feed 28 days	16S rRNA gene sequencing	excreta	No significant alteration of the composition or diversity of the microbiota	[148]
Rat	OTA: 70, 210 μg/kg BW 28 days	16S rRNA gene sequencing	excreta	Reduced within-subject diversity of the microbiota Increased relative abundance of *Lactobacillus* Reduced relative abundance of *Bacteroides*, *Dorea*, *Escherichia*, *Oribacterium*, *Ruminococcus*, and *Syntrophococcus*	[143]
Mice	AFB1*: 100, 160, 400 μg/kg BW 60 days	16S rRNA gene sequencing	intestinal contents (from jejunum to rectum)	*Lactobacillus* and *Bacteroides* are the dominant flora Differences in the relative abundance of bacterial flora Effects are not dose-dependent	[149]
Mice	DON: 1, 5 mg/kg BW 14 days	16S rRNA gene sequencing	excreta	Variation of microbiota composition with time *Bacteroidetes*, *Firmicutes*, *Proteobacteria* and *Verrucomicrobia* are the dominant bacterial phyla Reduced relative abundance of *Bacteroidaceae* family and *Alistipes* genus on day 14.	[150]
Mice	DON: 1, 5 mg/kg BW 14 days	shotgun sequencing	cecal digesta	The most abundant genera were *Lactobacillus*, *Mastadenovirus*, *Bacteroides*, *Mucispirillum*, and *Parabacteroides* Increased relative abundance of *Firmicutes* at low doses Increased relative abundance of *Bacteroidetes* at high doses	[151]
Mice	DON: 10 μg/kg BW 280 days	16S rRNA gene sequencing	excreta	Bacteria of the *Firmicutes* phyla are the most abundant Increase of *Deferribacteres*, *Proteobacteria*, TM7, *Verrucomicrobia*, *Tenericutes*, and *Cyanobacteria*. Reduced abundance of *Actinobacteria* and *Bacteroidetes* Several significant differences in taxonomic abundances at the family and genus levels.	[152]
Mice	ZEN: 10 mg/kg BW 14 days	16S rRNA gene sequencing	colon digesta	*Firmicutes*, *Bacteroidetes*, *Proteobacteria*, and *Actinobacteria* were the dominant phyla in the colon Reduced diversity of the microbiota Reduced abundance of *Firmicutes, Bacteroidetes*	[153]
Rabbit	ZEN: 400, 800, 1600 µg/kg BW 28 days	16S rRNA gene sequencing	cecal digesta	Reduced abundance of *Actinobacteria* and increase the abundance of *Cyanobacteria*, *Synergistetes*, and *Proteobacteria*. Reduced abundance of *Adlercreutzia*, *Blautia*, *Desulfitobacter*, *Lactobacillus*, *Oxalobacter*, and *p-75-a5*.	[154]
Rabbit	DON: 1.5 mg/kg BW 24 days	16S rRNA gene sequencing	ileal, cecal and colon digesta	Reduced abundance and diversity of the microflora, *Firmicutes*, *Bacteroidetes*, and *Proteobacteria* were the dominant phyla. Reduced relative abundance of *Proteobacteria*, *Actinobacteria*, and *Cyanobacteria* in both the ileum and caecum and increased relative abundance of *Firmicutes* and *Bacteroidetes* in the ileum and colon.in the ileum and Increased relative abundance of *Ruminococcaceae*, *Bacteriods*, and *Lachnospiraleaes* in the ileum, caecum, and colon. *Ruminococcaceae* represented the largest number of bacteria in the three intestinal segments at the genus level.	[155]
Pig	DON: 2.5 mg/kg feed 28 days	Bacterial culture Capillary electrophoresis	excreta	Variation of total aerobic bacterial flora with time Increase in total aerobic mesophilic bacteria max on day 7	[156]
Pig	DON: 1, 3 mg/kg feed 28 days	16S rRNA gene sequencing	small intestinal lumen digesta	*Firmicutes*, *Proteobacteria*, *Cyanobacteria* and *Actinobacteria* were the dominant phyla Reduced abundance of *Firmicutes* and increased abundance of *Actinobacteria* in duodenum and ileum Reduced abundance of *Proteobacteria* and increased abundance of *Cyanobacteria* in duodenum, jejunum, and ileum *Lactobacillus*, *Cupriavidus*, *Acinetobacter*, *Burholderia*, *Staphylococcus*, *Ochrobactrum*, *Corynebacterium*, and *Streptococcus* were the predominant generaReduced abundance of *Lactobacillus* and *Cupriavidus* and increased abundance of *Staphylococcus* Reduced abundance of *Burkholderia* in the duodenum and jejunum, but increased abundance in the ileum	[157]
Pig	DON: 0.61, 1.28, 2.89 mg/kg feed 28 days	16S rRNA gene sequencing	cecal digesta	Reduced abundances of unclassified f_Lachnospiraceae, Phascolarctobacterium and Ruminococcaceae_UCG-014 Increased Prevotella_9 and norank f_Prevotellaceae	[158]
Pig	ZEN: 40 μg/kg BW DON: 12 μg/kg BW ZEN + DON: 40 + 12 μg/kg BW42 days	EcoPlate tests	ascending colon digesta	Variation of total aerobic bacterial flora with time Same effect in nature whatever the toxin Lactic acid bacteria predominant floraDecrease in the number of mesophilic aerobic bacteria Decrease in the level of *C. perfringens*, *E. coli*, and *Enterobacteriaceae* family	[159]
Pig	ZEN: 0.8 mg/kg feed DON: 8 mg/kg feed 7 days	16S rRNA gene sequencing	colon digesta	*Firmicutes* and *Bacteroidetes* were the dominant phyla *Lactobacillus*, *Megasphaera*, and *Faecalibacterium* genera, and the unclassified *Clostridiaceae* family were the most abundant *Lactobacillus* was particularly more abundant in the DON (7.6%) and ZEN (2.7%) groups than in the control (0.2%).	[160]
Pig	ZEN: 5, 10, 15 µg/kg BW 7, 21, 42 days	Bacterial culture	duodenal cap, third duodenum part, jejunum, caecum, descending colon digesta	Microbial counts, mainly *E. coli* and *Enterococcus faecalis*, varied from the proximal to the distal segments of the intestinal tract ZEN affected the colony counts of microbiota rather than diversity Increased yeast and mold counts in all intestinal segments, in particular in the colon	[161]
Pig	DON + ZEN: 3.02 + 0.76 mg/kg feed 7 days Repeated exposure	16S rRNA gene sequencing	excreta	Reduced relative abundances of *Ruminococcaceae*, *Streptococcaceae*, and *Veillonellaceae* and increased *Erysipelotrichaceae* Microbiota returned to the initial state within 3 weeks after the end of a single or repeated DON/ZEN challenge	[162]
Pig	FB1+FB2: 8.6 + 3.2 mg/kg feed 63 days	Capillary single-stranded conformation polymorphism analysis	excreta	Variation of total aerobic bacterial flora with time Reversible alteration of the microbiota balance	[163]
Pig	FB1: 12 mg/kg feed 0, 8, 15, 22, 29 days	16S rRNA gene sequencing	excreta	Decrease in the diversity index, and shifts and constraints in the structure and the composition of the microbiota after 15 days of exposure that reached maximum after 22 days of exposure Increased *Lactobacillus* and reduced *Lachnospiraceae*, *Veillonellaceae* families, and particularly the genera *Mitsuokella*, *Faecalibacterium*, and *Roseburia*	[164]
Broiler	AFB1: 40 μg/kg feed 21 days	Bacterial culture	ileal digesta	No effect on *Lactobacilli*, *Bifidobacteria*, *C. perfringens*, *E. coli*	[165]
Broiler	Aflatoxins 0.5, 2 mg/kg feed 7 and 28 days	Bacterial culture	ileal digesta	Increased *E. coli*, *Salmonella*, *Klebsiella*, and total Gram- bacteria at day 28 of exposure Changes persisted for 14 days after exposure stopped	[166,167]
Broiler	AFB1: 1, 1.5, 2 mg/kg feed 21 days	Bacterial culture	cecal digesta	Increased total aerobic bacteria, total Gram - bacteria, variable effect on total lactic acid bacteria Effects are not always dose-dependent	[168]
Broiler	DON: 2.5, 5 and 10 mg/kg feed 35 days	16S rRNA gene sequencing	cecal digesta	Increased relative abundance of *Firmicutes* (decreased *Oscillospira*, *Clostridiaceae* genus, *Clostridium*, and *Ruminococcaceae* genera but increased *Clostridiales* genus) Reduced relative abundance of *Proteobacteria*	[169]
Broiler	FB1 + FB2: 10.4 + 8.2 mg/kg feed 15 days	16S rRNA gene sequencing	ileal digesta	Reduced abundance of *Candidatus* Savagella and *Lactobaccilus* spp., increased total *Clostridium perfringens*	[170]
Turkey	OTA: 199 to 462 µg/kg feed 21, 42, 63, 105 days	Bacterial culture	jejunum and cecal digesta, excreta	Reduced *Lactobacillus* spp. and *Bifidobacterium* spp. in samples of the intestinal content and the excreta after 15 weeks	[171]
Duck	OTA: 235 µg/kg BW 14 days	16S rRNA gene sequencing	excreta	Increased *Bacteroidetes* (phylum level), *Bacteroides* (genus level), *Bacteroides plebeius* (species level)	[172]

* calculated from data provided by the authors.

## Abbreviations

deoxynivalenol = DON; de-epoxy DON = DOM-1; nivalenol = NIV; diascetoxyscirpenol = DAS; monoacetoxyscirpeneol = MAS; acetyldeoxynivalenol = ADON; fusarenone X = FUS-X; aflatoxin B1 = AFB1; aflatoxin M1 = AFM1; ochratoxin A = OTA; ochratoxin α = OTα; zearalenone = ZEN; α-zearalenol = AZOL; β-zearalenol = BZOL; fumonisin B1 = FB1; gut-associated lymphoid tissue = GALT; body weight = BW; antimicrobial peptides = AMPs.
